# Retinal phototoxicity after macular hole surgery in a patient under paclitaxel

**DOI:** 10.1186/s12886-023-03086-x

**Published:** 2023-08-01

**Authors:** José M Ruiz-Moreno, María García‑Zamora, Jorge Ruiz‑Medrano

**Affiliations:** 1 Puerta de Hierro-Majadahonda University Hospital, C/ Manuel de Falla, 1, Majadahonda (Madrid), 28222 Spain; 2 Department of Ophthalmology, Castilla La Mancha University, Albacete, Spain; 3 Ocular Microsurgery Institute (IMO), Miranza Corporation, Madrid, Spain

**Keywords:** Macular phototoxicity, Paclitaxel, Vitrectomy

## Abstract

**Background:**

The purpose is to report the second case, to our knowledge, of suspected paclitaxel-induced phototoxic maculopathy following pars plana vitrectomy surgery.

**Case presentation:**

63-year-old phakic female who underwent an uneventful phaco-vitrectomy to treat a complete macular hole, developing macular phototoxicity in the post-operatively period that could not be explained by the surgery itself and could only be attributed to a possible photosensitization induced by the previous use of paclitaxel.

**Conclusions:**

The use of paclitaxel has been widely extended as a chemotherapy drug to treat breast cancer. It works by altering the intracellular microtubular reorganization and, based on this mechanism of action, photosensitivity has been previously described. We report a case of suspected paclitaxel-induced macular phototoxicity following ocular endoillumination during vitrectomy surgery.

## Introduction

It is known that light can cause significant visual loss by three possible mechanisms: thermal, mechanical or chemical. During pars plana vitrectomy (PPV) surgery, photothermal and photomechanical damage can be minimized using known, safe wavelength parameters [1]. However, there is little we can do to prevent retinal photochemical damage as it can occur under normal lighting conditions [2].

In the presence of some pharmacological agents, small amounts of solar or artificial radiation can cause retinal photochemical damage as a result of the release of oxygen-free radicals from the interaction between light and drug deposits in the retina [1, 3]. Patients exposed to photosensitizing agents are at risk of phototoxic injury during intraocular surgery.

Cases of maculopathy induced by paclitaxel have been previously reported [4–9]. A very similar case to the one reported in this article can be found in the literature, on a presumed paclitaxel-induced phototoxic maculopathy secondary to endo-illumination, causing unilateral vision loss following PPV in a 62-year-old woman receiving chemotherapy with paclitaxel [10].

## Case report

A 63-year-old-caucasian woman attended our clinic in December 2022 due to loss of vision in her left eye (OS). She presented ocular history of glaucoma under treatment in both eyes and history of breast cancer, treated with letrozole (aromatase inhibitor) and having previously received paclitaxel (taxane) until May 2021, pertuzumab (anti-HER2 antibody) and trastuzumab (anti-HER2 antibody) until May 2022.

**Fig. 1:**
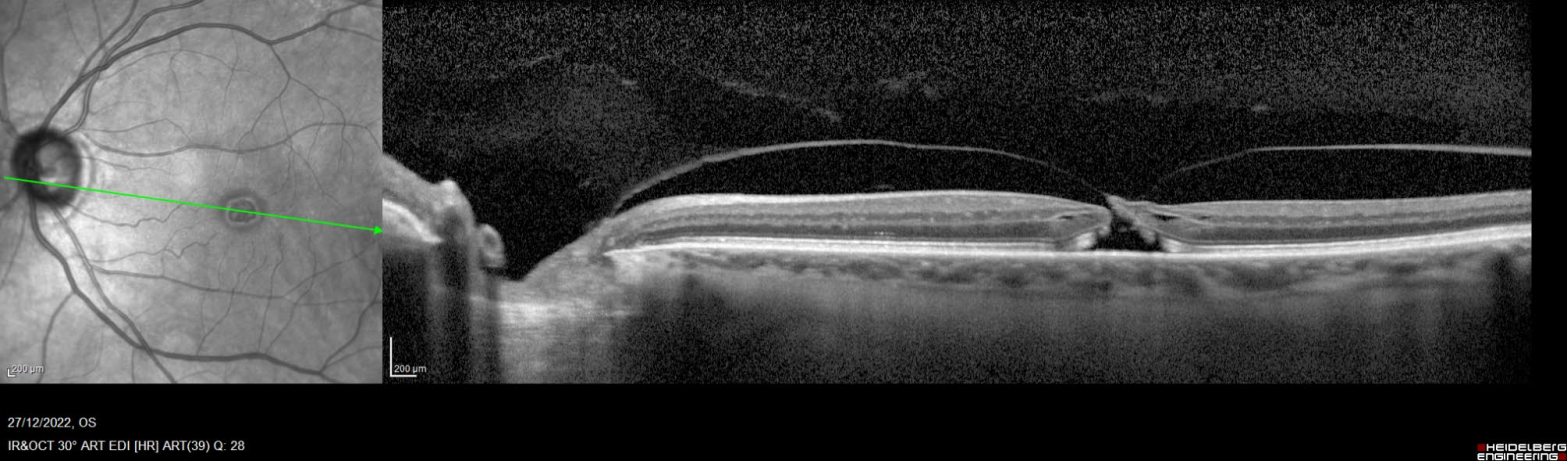
SD-OCT showing a stage II full-thickness macular hole with traction of the posterior hyaloid at the edge of the hole

Best corrected Visual Acuity (BCVA) in OS was 20/60; intraocular pressure (IOP) under topical treatment with Duotrav® (travoprost and timolol) was 18 mmHg in both eyes. Biomicroscopically, she showed a corticonuclear cataract in her OS. The ocular fundus study and spectral domain ocular coherence tomography (SD-OCT) revealed a full thickness stage II macular hole (MH) with tractional component (Fig. 1).

Combined cataract phacoemulsification and PPV was planned, being performed in January 2023 without any intraoperative incidents under local (retrobulbar) anesthesia. To perform the surgery, the EVA Nexus system (DORC Co., The Netherlands) was used placing the three 23-gauge trocars and performing the phacoemulsification and insertion of an Envista (Bausch &Lomb Co., Spain) intraocular lens, with a duration of 19:07 min. After that, central and peripheral PPV were performed assisted by the manufacturer’s stock light probe and using standard endo-illumination vitrectomy settings. Detachment of the posterior hyaloid after impregnation with triamcinolone, and internal limiting membrane peeling after staining with Membrane Blue Dual (DORC Co., The Netherlands) dye during 2 min without light followed. Internal fluid-air exchange and tamponade with 12% C3F8 completed the surgery with a total duration of 59:12 min without complications.

**Fig. 2:**
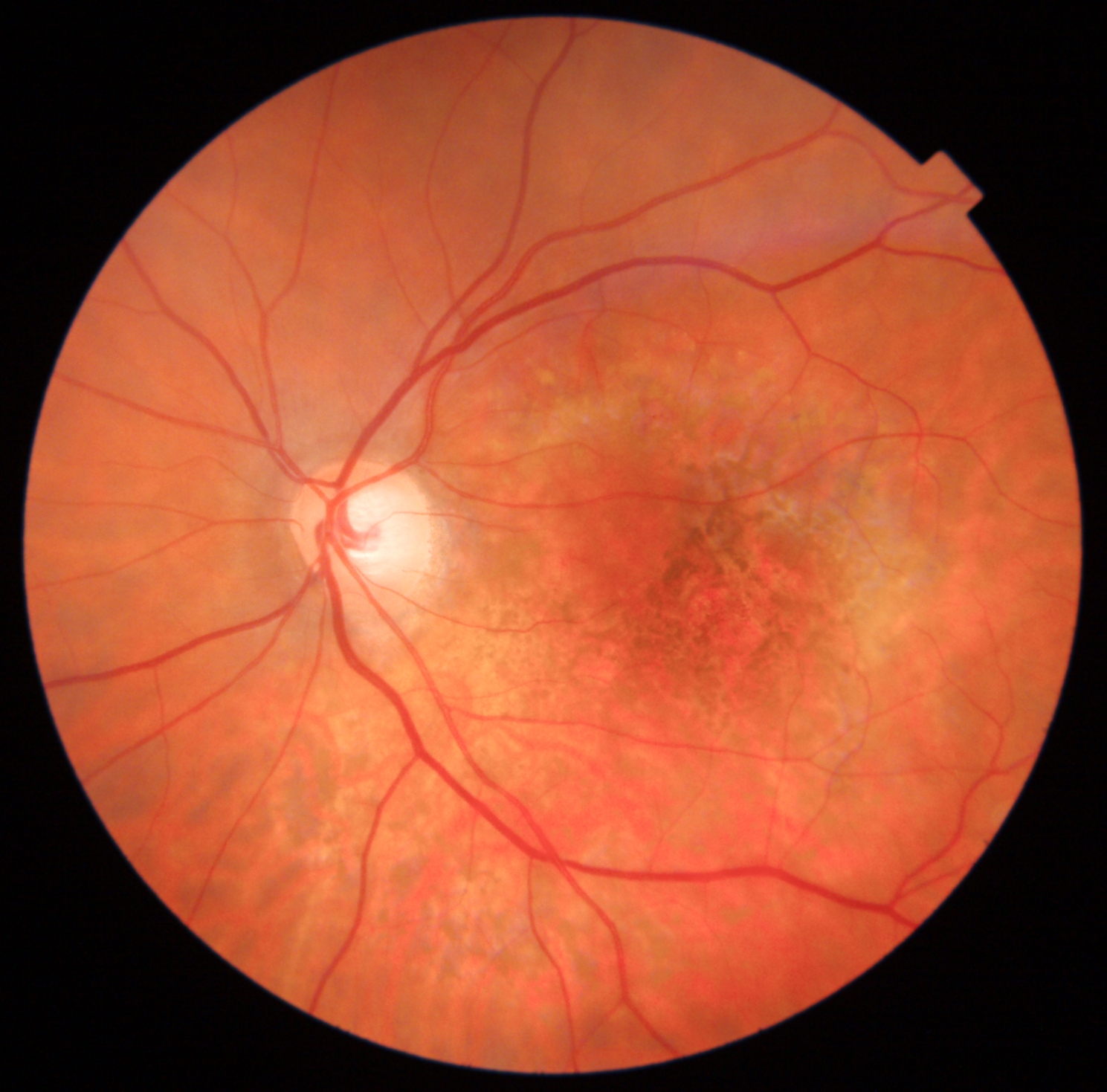
Color fundus photography in the early postoperative period showing pigmentary changes of the inferior area of the macula in the left eye

**Fig. 3:**
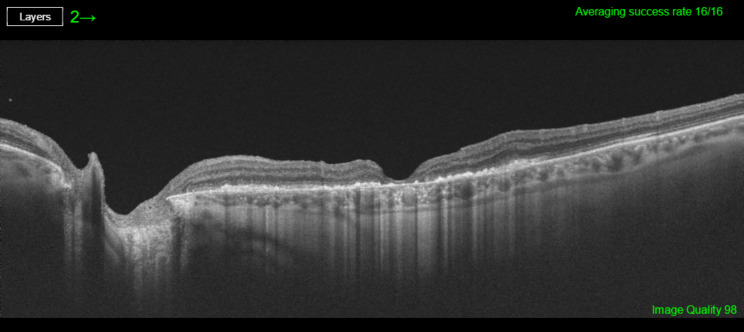
Postoperative SD-OCT showing a complete closure of the macular hole with significant alterations in the outer retinal layers

**Fig. 4:**
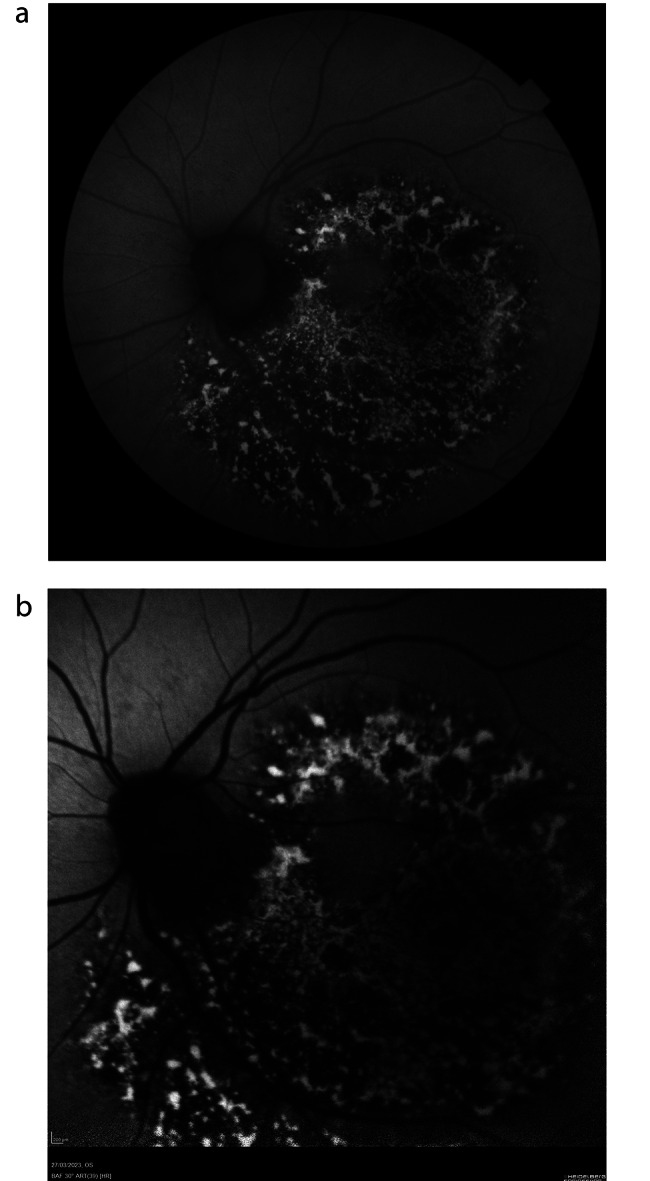
**(A)** Fundus autofluorescence (Triton OCT) image demonstrating patchy hyperautofluorescence and hypoautofluorescence secondary to RPE changes in the area exposed to endo-illumination. **(B)** Fundus autofluoresencence (Spectralis OCT) image confirming the phototoxicity

The postoperative period evolved without any notable incidents keeping prone position for 7 days and presenting normal IOP values. However, the patient reported from the first day the presence of a “black spot in the center of her visual field”. When the gas bubble disappeared BCVA in her OS was 20/400. After slit-lamp examination, pseudophakia was correct, while fundoscopy revealed retinal pigment epithelium (RPE) changes in the area that was illuminated by the endoprobe (Fig. 2); OCT showed a complete closure of the MH (Fig. 3) with alterations in outer retina, and fundus autofluorescence (FAF) testing revealed significant alteration of RPE in that area using both Triton, (Topcon Co, Japan; Fig. 4A) and blue FAF Spectralis platforms (Heidelberg Co, Germany; Fig. 4B).

**Fig. 5:**
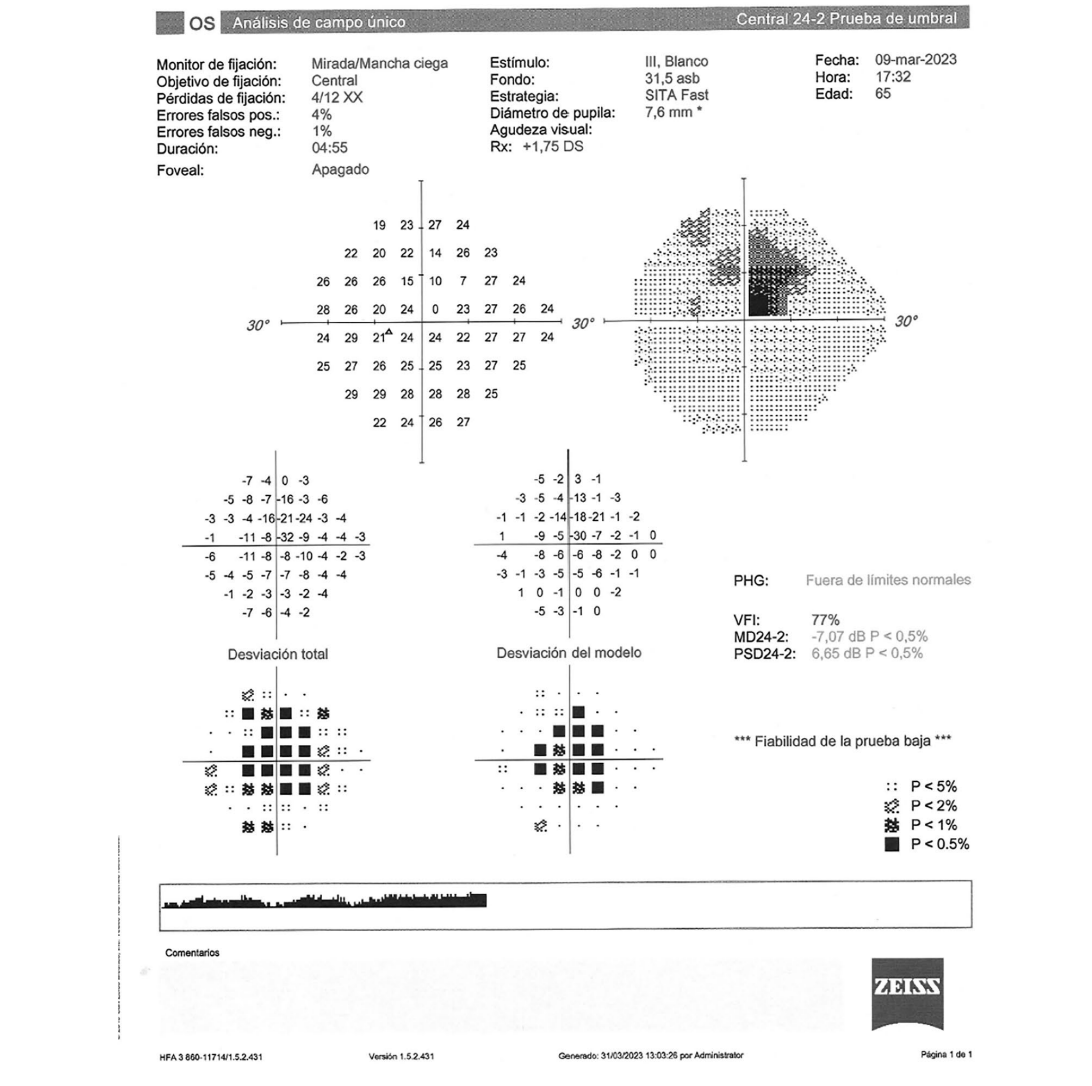
Visual Field showing an absolute central scotoma

**Fig. 6:**
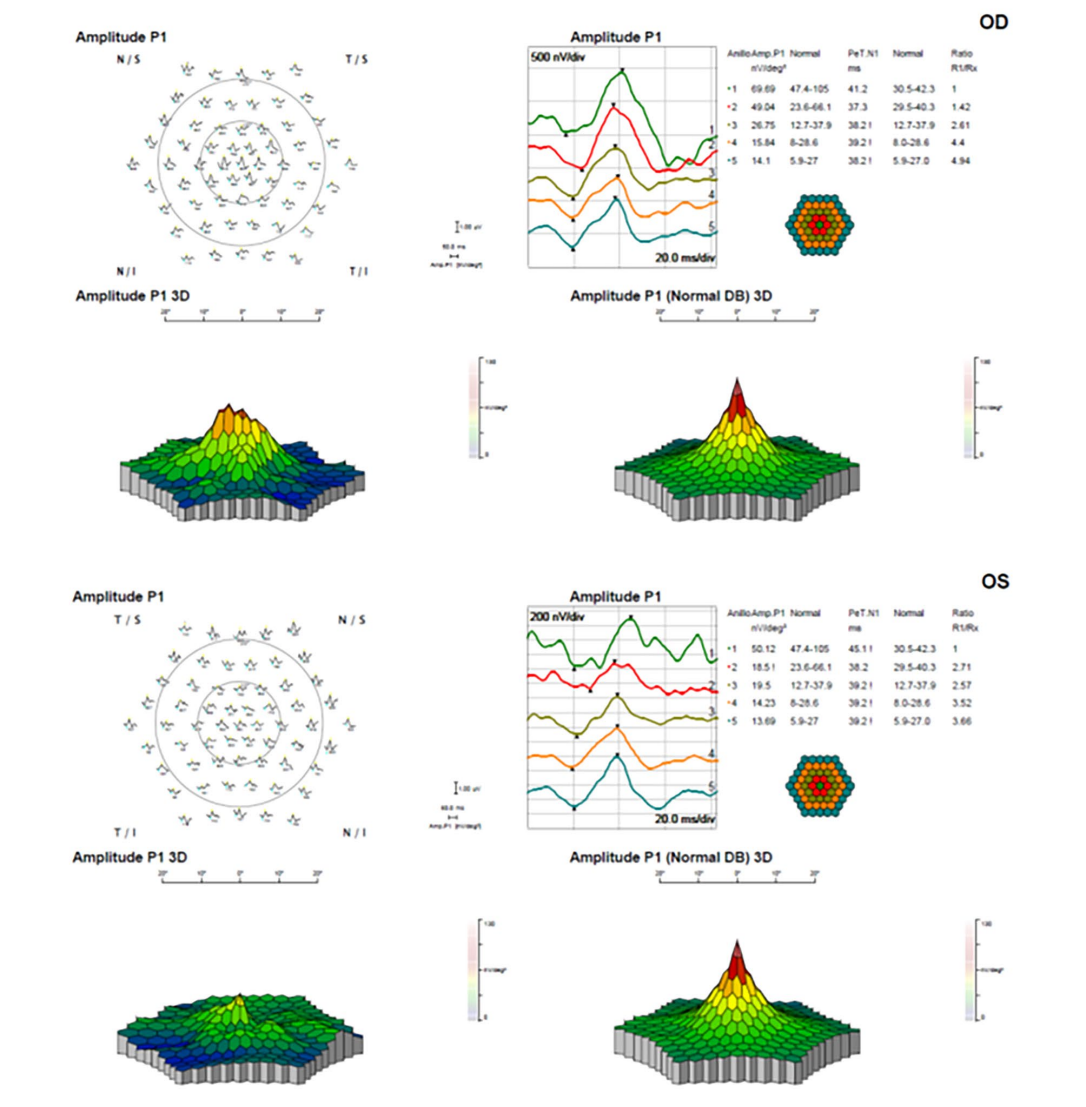
**(A)** and **(B)**: ERGmf showing a decrease in amplitudes in the affected area of the left eye compared to the right eye

The visual loss was further assessed by performing a visual field study (Humphrey Field Analyzer 3, Zeiss Co., Germany) demonstrating a central absolute scotoma (Fig. 5) and a multifocal electroretinogram (ERG) (Reti-report/scan 21, Roland Consult Co., Germany) (Fig. 6A and B) with a marked loss of sensitivity when compared to the right eye (OD).

The diagnosis of macular phototoxicity was established based on all these findings. Two months later, the patient reported a slight improvement with BCVA reaching 20/200 OS; no changes on ocular images were found.

## Discussion

Once suspected macular phototoxicity was diagnosed, the first decision taken at our center was to review the recording of the surgery, verifying a normal duration in absence of any unexpected intraoperative incident and standard lighting parameters. That same day four other PPV surgeries were performed using the same device, parameters, and batch of Membrane Blue Dual. The only potential causative agent of the reported maculopathy could be a photic injury induced by the combination of endo-illumination in the context of a photosensitization caused by one of the drugs used for the treatment of breast cancer. This hypothesis was supported by the fact that the same probe and parameters did not cause any consequence in the rest of the patients, while it did in the reported case.

Macular injury due to photothermal and photomechanical damage is unlikely in this patient, since surgical time and parameters were within normal ranges. The most likely explanation is a macular photochemical damage secondary to the use of a pharmacological agent that could be deposited in the RPE or neurosensory retinal cells. To our knowledge, none of the patient´s comorbidities (glaucoma, scoliosis, and osteoarthritis) nor the current medications (letrozole and duotrav) are associated with retinal phototoxicity. The induced maculopathy described is suspected to be a consequence of a phototoxicity injury produced during endo-illumination due to previous photosensitization of the retina by the previous use of paclitaxel. Although phototoxicity secondary to Brilliant Blue G (BBG) staining during vitrectomy has been previously reported in cases with a prolonged endo-illumination time, this could not be the case, as surgical time was significantly shorter compared with the cases reported in the published series and Membrane Blue Dual dye, containing 0.025% BBG only, was the dye used in this surgery [11–13].

In the absence of an alternative explanation, paclitaxel phototoxic maculopathy was considered as a plausible cause. Paclitaxel is known to induce maculopathy and the central and the inferior distribution of this maculopathy corresponds with the endo-illuminated area, explained by the probe entering superiorly, as happened in the other case reported in the literature [10].

Paclitaxel is a taxane, a chemotherapeutic drug that acts by altering intracellular microtubule reorganization. It is used for the treatment of breast and ovarian carcinoma. Reported ophthalmic side-effects include reduced visual acuity, scintillating scotomas and abnormal visual evoked potentials [4]. Paclitaxel-induced maculopathy has been described as causing macular edema, which was not present in our case, and is considered a long-term adverse effect [5].

Although the exact mechanism of paclitaxel-induced maculopathy remains unclear, several hypotheses have been proposed. Nakao et al. postulate that macular edema is a result of intracellular fluid accumulation caused by Muller cell dysfunction, as supported by delayed and reduced B-wave amplitudes on ERG [8]. A case of irreversible maculopathy induced by paclitaxel with macular edema has been previously reported with the authors stating that paclitaxel toxicity resulted in permanent cell damage [9]. To the best of our knowledge, this would be the second case of possible paclitaxel-induced maculopathy in the absence of macular edema, but unlike in the previous case, our patient had discontinued the drug 19 months prior to the surgery so there must have been a long-term deposit of the drug in the retinal cells.

Systemic use of paclitaxel can cause bilateral maculopathy; however, our patient suffered lesions in the eye exposed to the endo-illumination during the surgery only [5]. A likely explanation is that paclitaxel photosensitizes the RPE and photoreceptor cells, increasing the risk of photochemical damage during surgery. The mechanism could be related to taxane-induced dermal photosensitivity, which is thought to be caused by alterations in porphyrins [14]. Since porphyrins have been linked to ocular lesions, including retinal RPE phototoxicity, it is possible that the alterations of porphyrins due to the use of paclitaxel may cause photosensitization of the RPE [2].

## Conclusions

This is the second clinical case of suspected paclitaxel-induced macular phototoxicity and the first case arising after the drug had been previously discontinued. With the observed phototoxic effects of paclitaxel on the macula, its concomitant or previous use may contribute to a poor prognosis after vitrectomy surgery and should be dully considered in future possible cases.

## Data Availability

The datasets used and/or analysed during the current study available from the corresponding author on reasonable request.

## References

[1] Glickman RD (2002). Phototoxicity to the retina: mechanisms of damage. Int J Toxicol.

[2] Boulton M, R´ozanowska M, R´ozanowski B (2001). Retinal photodamage. J Photochem Photobiol B Biol.

[3] Joshi KM, Gill MK (2017). Amiodarone: a potential risk factor for retinal phototoxicity. Am J Ophthalmol Case Rep.

[4] Hofstar LS, de Vries EGE, Willemse PHB (2002). Ophthalmic toxicity following paclitaxel infusion. Entomol Exp Appl.

[5] Kuznetcova TI, Cech P, Herbort CP (2012). The mystery of angiographically silent macular oedema due to taxanes. Int Ophthalmol.

[6] Joshi MM, Garretson B (2009). Paclitaxel Maculopathy.

[7] Meyer KM, Klink T, Ugurel S, Br¨ocker EB (2012). Regression of paclitaxel-induced maculopathy with oral acetazolamide. Graefe’s Arch Clin Exp Ophthalmol.

[8] Nakao S, Ikeda Y, Emi Y, Ishibashi T (2016). Possibility of müller cell dysfunction as the pathogenesis of paclitaxel maculopathy. Ophthalmic Surg Lasers Imag Retin.

[9] Amigo MHL, Falabella P, Bettarello ˆA, Ghirelli W (2015). Irreversible visual loss after use of Paclitaxel. Rev Bras Oftalmol.

[10] Malcolm J, Oi Lune Wong C, Ching J, Saidkasimova S. Paclitaxel may be a risk factor for retinal phototoxicity. Am J Ophthalmol Case Rep. 2022; 25101292. 10.1016/j.ajoc.2022.101292 PMC8790282 35112024

[11] Soni A, Parameswarappa DC, Tyagi M et al. Brilliant blue G toxicity in macular hole surgeries: a report on combined phototoxicity and dye-induced macular damage. Semin Ophthalmol. 2021;36. 10.1080/08820538.2021.1928717 34010087

[12] Venkatesh R, Aseem A, Jain K, Yadav N (2017). Combined brilliant blue G and xenon light induced outer retinal layer damage following macular hole surgery. Indian J Ophthalmol.

[13] Federico O, Anniken B-J, Carlos M. Diffuse retinal pigment epithelium atrophy following pars plana vitrectomy for high myopic macular hole assisted by Brilliant Blue G: a case report. Am J Ophthalmol Case Rep. 2021;23. 10.1016/j.ajoc.2021.101148 PMC8246242 34222716

[14] Sibaud V, Leboeuf NR, Roche H (2016). Dermatological adverse events with taxane chemotherapy. Eur J Dermatol.

